# Engineering thermophilic *Geobacillus thermoglucosidasius* for riboflavin production

**DOI:** 10.1111/1751-7915.13543

**Published:** 2020-02-25

**Authors:** Zhiheng Yang, Qingqing Sun, Gaoyi Tan, Quanwei Zhang, Zhengduo Wang, Chuan Li, Fengxian Qi, Weishan Wang, Lixin Zhang, Zilong Li

**Affiliations:** ^1^ State Key Laboratory of Bioreactor Engineering East China University of Science and Technology Xuhui District Shanghai 200237 China; ^2^ State Key Laboratory of Microbial Resources Institute of Microbiology Chinese Academy of Sciences Chaoyang District Beijing 100101 China

## Abstract

The potential advantages for fermentation production of chemicals at high temperatures are attractive, such as promoting the rate of biochemical reactions, reducing the risk of contamination and the energy consumption for fermenter cooling. In this work, we *de novo* engineered the thermophile *Geobacillus thermoglucosidasius* to produce riboflavin, since this bacterium can ferment diverse carbohydrates at an optimal temperature of 60°C with a high growth rate. We first introduced a heterogeneous riboflavin biosynthetic gene cluster and enabled the strain to produce detectable riboflavin (28.7 mg l^−1^). Then, with the aid of an improved gene replacement method, we preformed metabolic engineering in this strain, including replacement of *ribC*_Gtg_ with a mutant allele to weaken the consumption of riboflavin, manipulation of purine pathway to enhance precursor supply, deletion of *ccpN*_Gtg_ to tune central carbon catabolism towards riboflavin production and elimination of the lactate dehydrogenase gene to block the dominating product lactic acid. Finally, the engineered strain could produce riboflavin with the titre of 1034.5 mg l^−1^ after 12‐h fermentation in a mineral salt medium, indicating *G. thermoglucosidasius* is a promising host to develop high‐temperature cell factory of riboflavin production. This is the first demonstration of riboflavin production in thermophilic bacteria at an elevated temperature.

## Graphical abstract

Taking advantage of *Geobacillus thermoglucosidasius* with the characteristics of fast growth and utilization of diverse inexpensive substrates, here we demonstrated that this strain could be used as a promising alternative host for biosynthesis of riboflavin. Afterward, a sequential of engineering strategy was applied to improve riboflavin titers in the *G. thermoglucosidasius* producer to 1034.5 mg l^−1^ at 12‐h flask fermentation in a simple mineral salt medium. As proof‐of‐concept, our work presented the feasibility of riboflavin production by engineering the thermophilic *G. thermoglucosidasius*. More importantly, the present work paves the way to further systematic metabolic engineer this thermophilic host for riboflavin production.

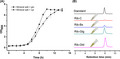

## Introduction

The advantages of using thermophilic bacteria as cell factory include reducing the risk of contamination in the bioreactors with ambient microorganisms, saving the energy consumption for fermenter cooling and accelerating the biochemical processes in fermentation. In addition, many glycolytic thermophiles are able to use polymeric or short oligomeric carbohydrates with low nutrient requirements. These merits collectively make thermophiles as favourable platform organisms in a sustainable bio‐based economy (Chen and Jiang, 2018). Thus, development of thermophilic bacteria is of great interest for biotechnology to generate useful products using a wide range of inexpensive substrates.

*Geobacillus thermoglucosidasius*, which belongs to a facultative anaerobic, rod‐shaped, Gram‐positive and endospore‐forming bacterium (Nazina *et al.*, 2001), tends to ferment a wide range of substrates, including both cellobiose and pentose sugars. *G. thermoglucosidasius* DSM2542 has been engineered and exploited for industrial bioethanol production from lignocellulosic feedstocks (Cripps *et al.*, 2009). This is primarily due to its rapid growth rate and ability to ferment a broad range of monosaccharides, cellobiose and short‐chain oligosaccharides (Taylor *et al.*, 2009). The already success in bioethanol production and the predominant characteristics make *G. thermoglucosidasius* DSM2542 as a potential workhorse for production of other value‐added chemicals, such as organic acids and amino acids. As a consequence, the whole genome of this strain has been sequenced recently to provide the genetic basis for the possibility of producing other chemicals via systematic metabolic engineering (Chen *et al.*, 2015). More importantly, this strain is amenable to genetic manipulation in spite of its difficulty (Cripps *et al.*, 2009). However, this strain has not been developed as cell factory for other value‐added chemicals until now, except for the purpose of producing biofuels including ethanol (Cripps *et al.*, 2009) and isobutanol (Lin *et al.*, 2014).

Riboflavin (vitamin B2) is the direct precursor of FMN and FAD, which are essential components of cellular physiology required by all bacteria, plants and animals. Riboflavin has been widely applied in many fields such as pharmaceuticals and cosmetics, as well as human and animal nutrition (Powers, 2003). Commercial riboflavin production now is achieved by fermentation using the Gram‐positive bacterium *Bacillus subtilis* or the hemiascomycete *Ashbya gossypii* breeded by a combination of classical mutagenesis, genome shuffling and rational metabolic engineering (Schwechheimer *et al.*, 2016). It is well established that two important precursors are responsible for riboflavin biosynthesis: ribulose 5‐phosphate deriving from the pentose phosphate (PP) pathway and guanosine triphosphate (GTP) originating from purine biosynthesis (Bacher *et al.*, 2000). In addition to the necessary precursors, a mass of ATP is also required for riboflavin biosynthesis; hence, a considerable amount of biological heat is inevitably generated during the fermentation. Since both *B. subtilis* and *A. gossypii* are mesophilic bacteria, the production process in industry consumes a large quantity of energy for fermenter cooling.

To save the energy consumption for fermenter cooling, here we endeavour to develop a thermophilic host *G. thermoglucosidasius* for riboflavin production. Since it could ferment a wide range of substrates with low nutrient requirements for fast growth, we presumed that this strain may equipped with robust PP and purine pathways, and consequently, the adequate ribulose 5‐phosphate and GTP precursors could also be supplied for riboflavin production. As a proof of concept, we indeed demonstrated that *G. thermoglucosidasius* DSM2542 could be used as a promising alternative producer of riboflavin in this work. Afterwards, we applied a sequential of engineering strategies to improve riboflavin titres in *G. thermoglucosidasius*. Finally, the titre of riboflavin was increased to 1034.5 mg l^−1^ in a simple mineral salt medium.

## Results

### *G. thermoglucosidasius* as a host for riboflavin production

**Figure 1:**
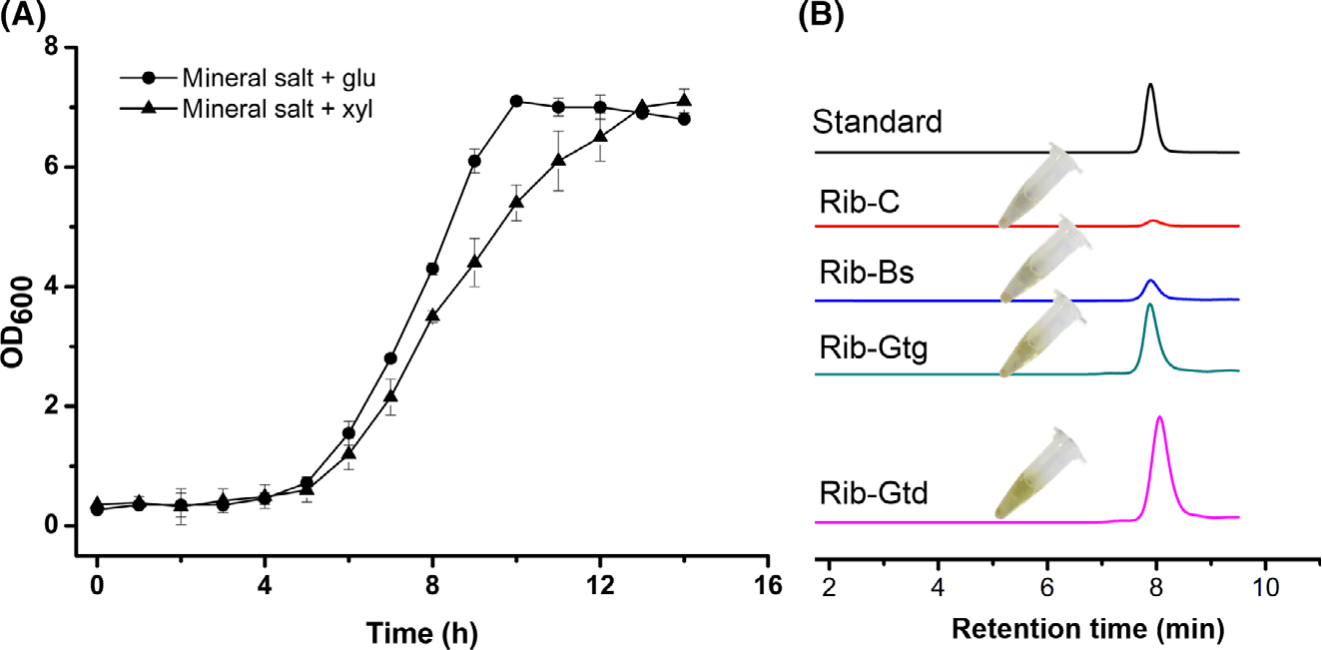
*G. thermoglucosidasius* DSM2542 as a host for riboflavin production. A. Growth curve of *G. thermoglucosidasius* DSM2542 in mineral salt medium with glucose or xylose as carbon source. B. Expression of native or heterogeneous *rib* cluster in *G. thermoglucosidasius* DSM2542 for riboflavin production. Data shown are the average and standard derivation (SD) of three independent experimental replicates.

To evaluate the growth characteristics of thermophilic *G. thermoglucosidasius* DSM2542*,* we determined the growth curves under various culture conditions. We observed a reproducible, vigorous growth with a maximum growth rate of 0.33 h^−1^ and reaching stationary within 6 h in 2SPY (Sheng *et al.*, 2017) liquid medium (Fig. S1). In parallel, we also tested the growth curves in mineral salts medium, which was advantageous in both the fermentation costs and the following purification steps (Wang *et al.*, 2011, 2011). We found that, either glucose or xylose as main carbon source, the growth rates are comparable, reaching stationary phase within 12 h (Fig. 1A). Since the capability of fast growth at 60°C with both hexose and pentose sugars, we presumed that *G. thermoglucosidasius* is qualified to provide building blocks and energy for riboflavin biosynthesis from central carbon metabolism and that this strain may be an alternative host to produce riboflavin without fermenter cooling.

We cloned the riboflavin biosynthetic gene clusters (*rib*) from *Bacillus subtilis* 168, *G. thermodenitrificans* NG80‐2 and the native host *G. thermoglucosidasius* DSM2542 into the pUCG3.8 plasmid and then these plasmids were expressed in *G. thermoglucosidasius* DSM2542 respectively. To our delight, we observed the yellow product in the strains with additional *rib* from *G. thermodenitrificans* NG80‐2 and *G. thermoglucosidasius* itself (Fig. 1B). Further, using HPLC, we confirmed that the yellow product indeed was riboflavin, indicating that the redundant intermediates of PP and GTP pathway of this strain could be used to biosynthesize riboflavin (Fig. 1B). The peak area of HPLC indicated that *G. thermoglucosidasius* with the *rib* from *G. thermodenitrificans* NG80‐2 gave the highest production (Fig. S2), we therefore used this *rib* in the following study.

### Development of a convenient DNA replacement method

To engineering the thermophilic host for further improvement of riboflavin production, the reliable manipulation of the genome of *G. thermoglucosidasius* was a prerequisite. A vector system used for *Geobacillus* spp. had been constructed previously (Taylor *et al.*, 2008; Cripps *et al.*, 2009). However, only one thermostable marker (kanamycin nucleotidyltransferase gene) is not convenient for the second‐round selection of double cross‐over, where usually need 2–5 sequential subcultures without kanamycin and then rely on vast screening by colony PCR to identify the required strains. For convenient selection without modifying the host genome, we tested several markers including the fluorescent protein sfGFP (Bai *et al.*, 2015), the visual reporter IdgS‐Sfp system (Xie *et al.*, 2017) and the catechol 2,3‐dioxygenase XylE (Wang *et al.*, 2015). We found that sfGFP was very stable in *G. thermoglucosidasius* at 60–70°C (Fig. S3A). Hence, sfGFP could be used as an ideal second‐selection marker in thermophilic host.

**Figure 2:**
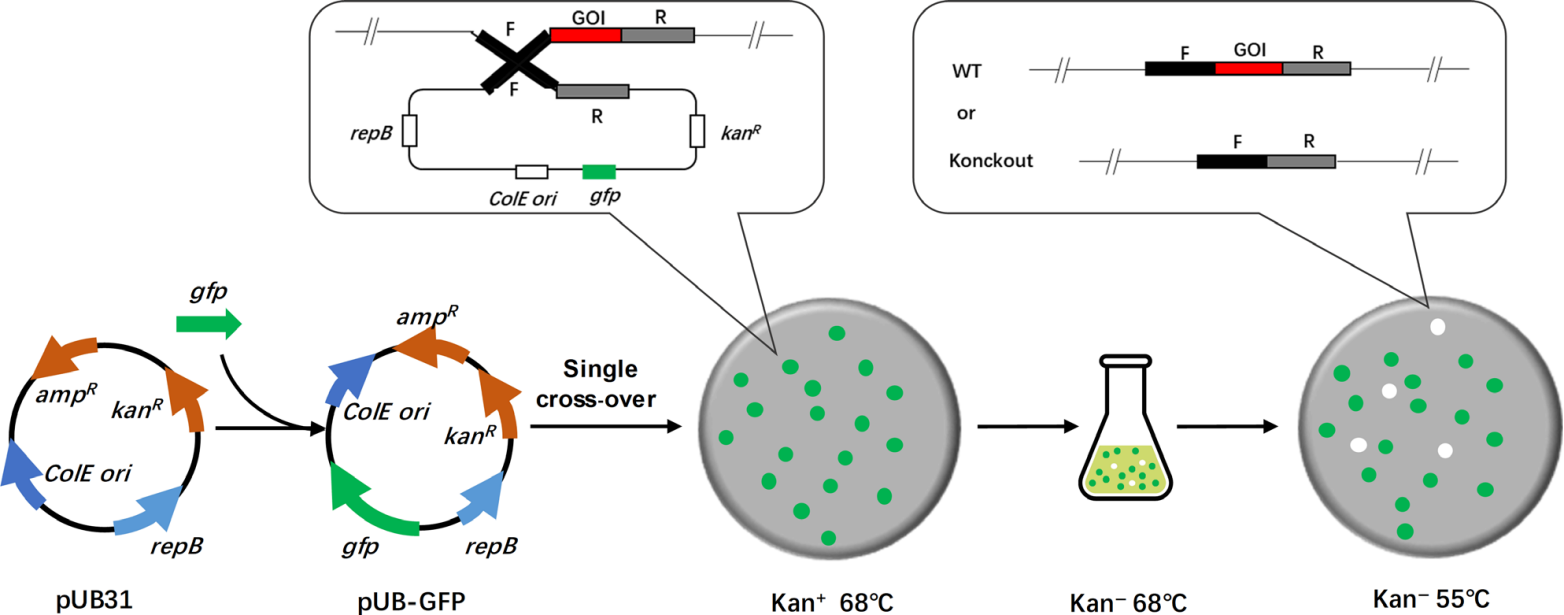
Schematic for the improved DNA replacement method. *amp*, ampicillin; *kan*, kanamycin; *gfp,* gene encoding green fluorescent protein; ColE ori, *Escherichia coli* origin of replication; *repB*, *Geobacillus* sp. origin of replication; F, upstream homologous arm; R, downstream homologous arm; GOI, gene of interest; and WT, wild‐type

Using the two markers, we developed a very convenient DNA replacement method in *G. thermoglucosidasius* (Fig. 2). Because the vector we used here was not capable of replication at 65°C and above, colonies in which the complete vectors had integrated into the genome through homologous recombination could be selected by both kanamycin resistance and green fluorescence at 68°C. Then, undergone only one sequential subcultures without kanamycin, the potential double cross‐over clones could be selected by the phenotype of fluorescence loss on the plate. To test the performance of this DNA replacement method, we deleted *pyrE* which encodes orotate phosphoribosyltransferase in *G. thermoglucosidasius* DSM2542, and confirmed that this method could observably identify those cells in which the integrated plasmid had been lost (Fig. S3A). We picked the clones without fluorescence and confirmed by PCR. The result indicated that approximately half clones were the desired mutants (Fig. S3B).

### Reducing the cellular consumption of riboflavin

**Figure 3:**
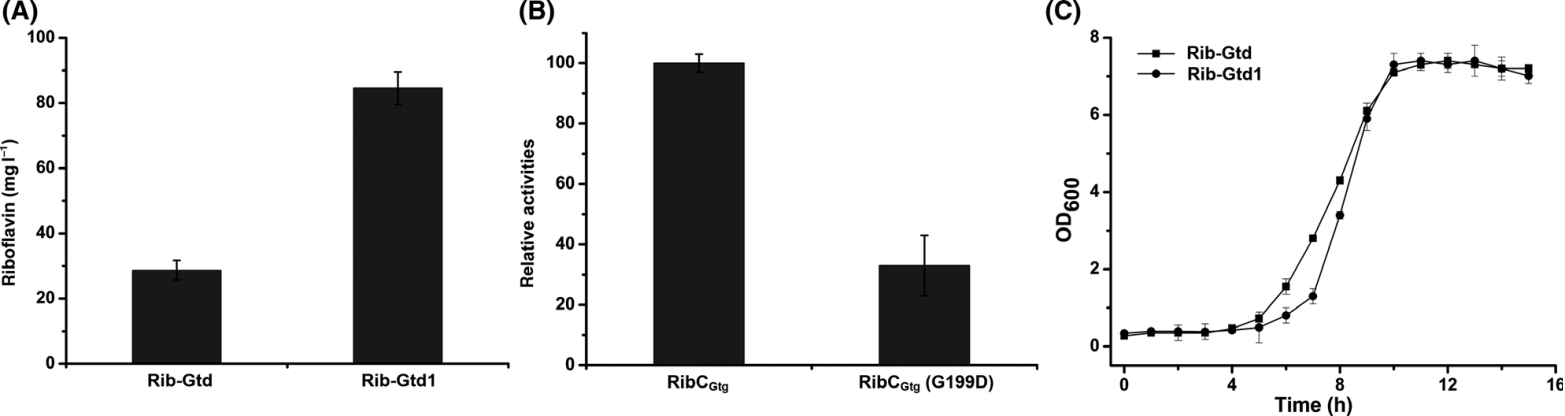
Reducing the cellular consumption of riboflavin. A. Riboflavin titre improvement when replacing the RibC_Gtg_ with RibC_Gtg_(G199D). B. Effect of the site mutant in RibC_Gtg_(G199D) on the activity of riboflavin kinase. C. Influence of the site mutant in RibC_Gtg_(G199D) on cell growth. Data shown are the average and SD of three independent experimental replicates.

Riboflavin is consumed to generate cofactors FMN and FAD *in vivo*, and hence, we needed to turn down the cellular consumption of riboflavin. It was reported that the G199D substitution of the bifunctional riboflavin kinase/FMN adenylyltransferase RibC could reduce 95% activity and resulted in low cellular consumption of riboflavin in *Bacillus subtilis* (Bresler *et al.*, 1973; Wang *et al.*, 2018). We aligned the RibC in *Bacillus subtilis* with the homologous protein encoded by *ribC*_Gtg_ (AOT13_09320) in *G. thermoglucosidasius* and found that they share 55% identities (Fig. S4). We therefore mutated the corresponding site of *ribC*_Gtg_ in *G. thermoglucosidasius* (Fig. S5). As expected, the titre of riboflavin with the desired allele exchange mutant (Rib‐Gtd1) increased 194.8% to 84.6 mg l^−1^ (Fig. 3A). Also, we measured the effect of this site mutant on the activity of riboflavin kinase. Result indicated that, compared with the native riboflavin kinase, the mutant showed 33% activity (Fig. 3B). To evaluate the influence of this mutant, we profiled the growth of both starting and mutant strains, and observed nearly identical growth curves (Fig. 3C).

### Manipulation of purine pathway to enhance precursor supply

**Figure 4:**
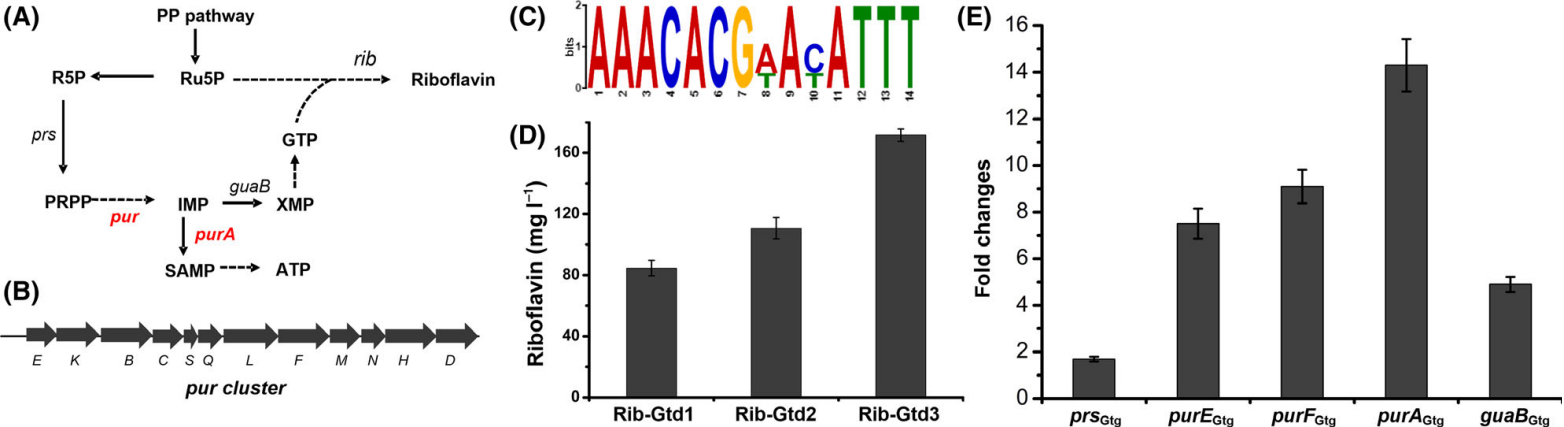
Manipulation of purine pathway to enhance precursor supply. A. Schematic overview of precursor pathways for riboflavin biosynthesis. R5P, ribose‐5‐phosphate; Ru5P, ribulose‐5‐phosphate; PRPP, 5‐phospho‐α‐d‐ribosyl‐1‐pyrophosphate; IMP, inosine 5′‐monophosphate; SAMP, succinyladenosine monophosphate; ATP, adenosine 5′‐triphosphate; XMP, xanthosine 5′‐monophosphate; GTP, guanosine 5′‐triphosphate; *prs*, encoding phosphoribosylpyrophosphate synthetase; *guaB*, encoding inosine monophosphate dehydrogenase; *and purA, encoding adenylosuccinate synthetase.* B. Schematic of the genes of purine operon (*pur*). C. Putative motif with high consistency to the PurR binding site (PurBox). D. Comparison of riboflavin production of the engineered mutant strains. E. Changes of the relative transcript levels of genes involving in purine pathway in the engineered strain with PurR_Gtg_ deletion. Data shown in (D) and (E) are the average and SD of three independent experimental replicates.

Biosynthesis of riboflavin needs GTP as precursor generating via purine pathway (Fig. 4A). Besides riboflavin, purine nucleotides are essential metabolites for living organisms because they are involved in many important processes, such as nucleic acid synthesis, energy supply and biosynthesis of several amino acids (Smith *et al.*, 1994). Hence, the pool of intracellular purine nucleotides is maintained under homeostasis and the *de novo* purine biosynthetic pathway is tightly regulated by transcription repression. It is reported that PurR regulates the transcription of all gene encoding enzymes for synthesis of IMP, AMP and GMP in *B. subtilis* (Sinha *et al.*, 2003). We searched the homologue of PurR of *B. subtilis* and found that AOT13_02815 (here named PurR_Gtg_) shared 71% identities with PurR (Fig. S6). Further, the promoter regions of purine operon (Fig. 4B), *purR*_Gtg_ and the *purA* homologue gene (AOT13_03190, *purA*_Gtg_) in *G. thermoglucosidasius* DSM2542 were submitted to MeMe suite (Bailey *et al.*, 2009) to identify the potential motifs. We found a conservative motif with high consistency to the PurR binding site (PurBox) of *B. subtilis* (Fig. 4C). These results collectively demonstrate that PurR_Gtg_ may inhibit purine pathway in *G. thermoglucosidasius*.

We deleted *purR*_Gtg_ in *G. thermoglucosidasius* Rib‐Gtd1 using our DNA replacement method to generate Rib‐Gtd2 (Fig. S7). By measuring the titre of riboflavin, we confirmed that Rib‐Gtd2 improved 30.8% of riboflavin titre to 110.7 mg l^−1^ (Fig. 4D). Moreover, we detected the transcription of purine pathway. Indeed, we observed a higher transcription level of the genes involving in purine pathway in the strain Rib‐Gtd2 (Fig. 4E), indicating that the purine pathway for both ATP and GTP biosynthesis was enhanced when deleted the repressor PurR_Gtg_.

However, the enhanced transcript level of *purA*_Gtg_ gene in *G. thermoglucosidasius* Rib‐Gtd2 could divert IMP to ATP (Fig. 4A and 4E), which is undesired for riboflavin production. To avoid the flux of purine pathway towards adenine, we further deleted *purA*_Gtg_ to generate the strain Rib‐Gtd3 (Fig. S8). Again, we improved the riboflavin titre to 171.6 mg l^−1^ in mineral salts medium at 12 h flask fermentation (Fig. 4D).

### Deletion of CcpN_Gtg_ to tune central carbon catabolism

**Figure 5:**
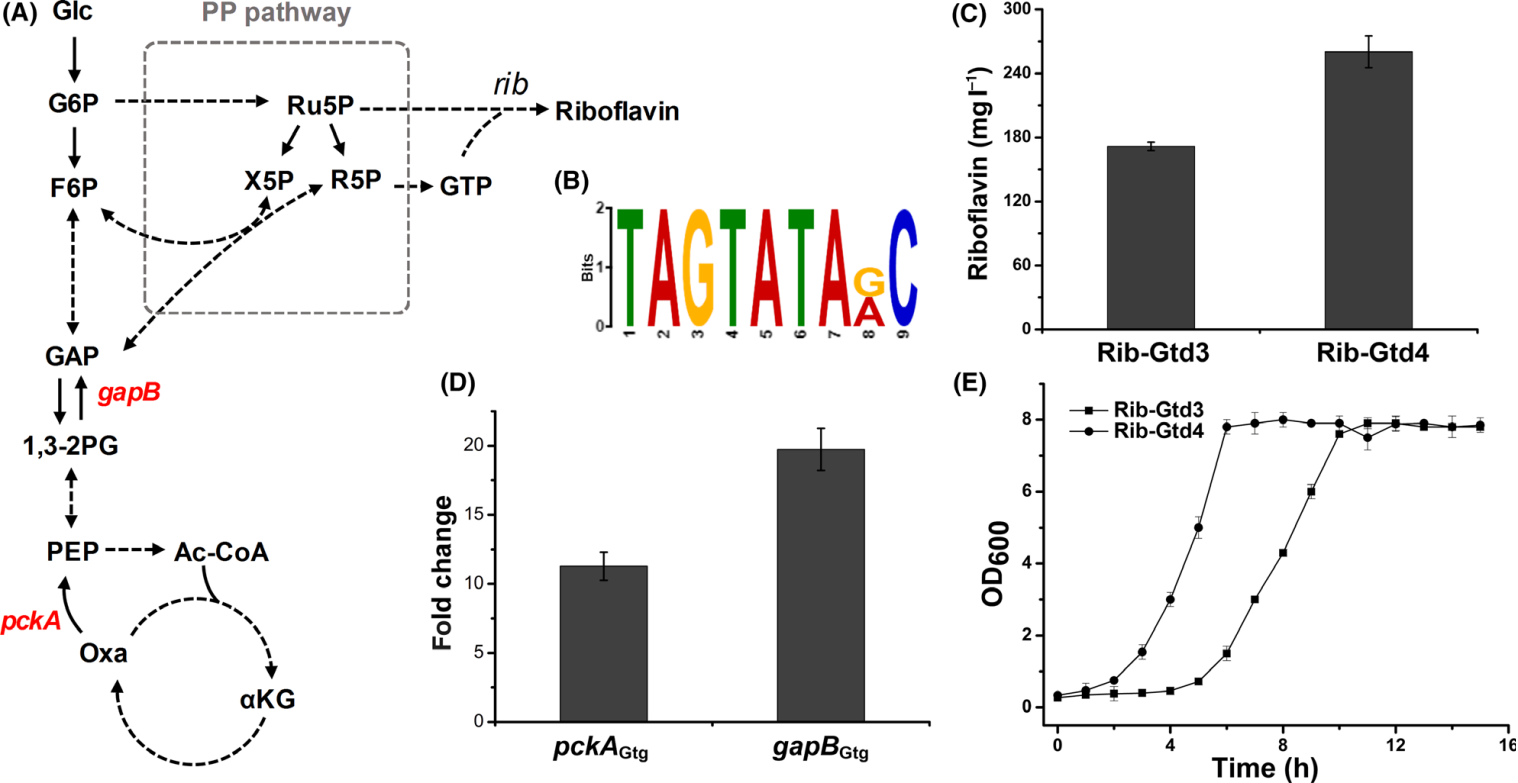
Deletion of CcpN_Gtg_ to tune central carbon catabolism. A. Schematic overview of central carbon metabolism and riboflavin biosynthesis. Glc, glucose; G6P, glucose 6‐phosphate; F6P, fructose‐6‐phosphate; GAP, glyceraldehyde‐3‐phosphate; 1,3‐2PG, 1,3‐bisphosphoglycerate; PEP, phosphoenolpyruvate; Oxa, oxaloacetate; Ac‐CoA, acetyl coenzyme A; αKG, oxoglutarate; X5P, xylulose‐5‐phosphate; *gapB,* encoding NADPH‐dependent glyceraldehyde‐3‐phosphate dehydrogenase; *and pcka,* encoding phosphoenolpyruvate carboxykinase. B. Putative motif with high consistency to CcpN binding site in *B. subtilis.* C. Deletion of CcpN_Gtg_ contributed to riboflavin production. D. Deletion of CcpN_Gtg_ enhanced the transcript levels of *pckA*_Gtg_ and *gapB*_Gtg_. E. Effect of the deletion of CcpN_Gtg_ on the cell growth. Data shown in (C), (D) and (E) are the average and SD of three independent experimental replicates.

PP pathway provides the two direct precursors ribulose 5‐phosphate and GTP for riboflavin biosynthesis directly or indirectly (Schwechheimer *et al.*, 2016). To tune central carbon catabolism towards PP pathway (Fig. 5A), we noted that the transcription regulator CcpN of *B. subtilis* had been recently characterized to reroute the main glucose catabolism from glycolysis to PP pathway (Tannler *et al.*, 2008). We therefore sought the homologue of this regulator in *G. thermoglucosidasius* and identified AOT13_16900 (herein named CcpN_Gtg_) sharing 75% identities (Fig. S9). This result inspired us to speculate that similar regulatory mechanism (Servant *et al.*, 2005) might occur in *G. thermoglucosidasius*, that is CcpN_Gtg_ may repress NADPH‐dependent glyceraldehyde‐3‐phosphate dehydrogenase gene (AOT13_18085, *gapB*_Gtg_) and the phosphoenolpyruvate carboxykinase gene (AOT13_18565, *pckA*_Gtg_). To verify this, we searched the conservative nucleotide sequences in the promoter region of these two target genes and extracted a perfectly conserved motif with 7 nt sequence (Fig. 5B), which agree well with the DNA binding site of CcpN in *B. subtilis*.

Next, we deleted *ccpN*_Gtg_ in *G. thermoglucosidasius* Rib‐Gtd3 using our DNA replacement method to construct engineered strain Rib‐Gtd4 (Fig. S10). Then, we monitored the riboflavin production. Results showed that riboflavin production of strain Rib‐Gtd4 increased from 171.6 ± 4 to 260.3 ± 15 mg l^−1^, which is 1.51‐fold higher than Rib‐Gtd3. Furthermore, we tested the transcription level of the two putative target genes by RT‐qPCR. As shown in Figure 5D, it presented that the transcript level of *gapB*_Gtg_ and *pckA*_Gtg_ was higher in the strain Rib‐Gtd4 compared with that in Rib‐Gtd3. To evaluate the effect of *ccpN*_Gtg_ deletion on the cell growth, we tested the growth curves of the strains Rib‐Gtd4 and Rib‐Gtd3. To our surprise, we observed that Rib‐Gtd4 grew faster at early stage, but the final biomass of both strains was nearly identical (Fig. 5E).

### Elimination of main competing pathways in carbon metabolism

**Figure 6:**
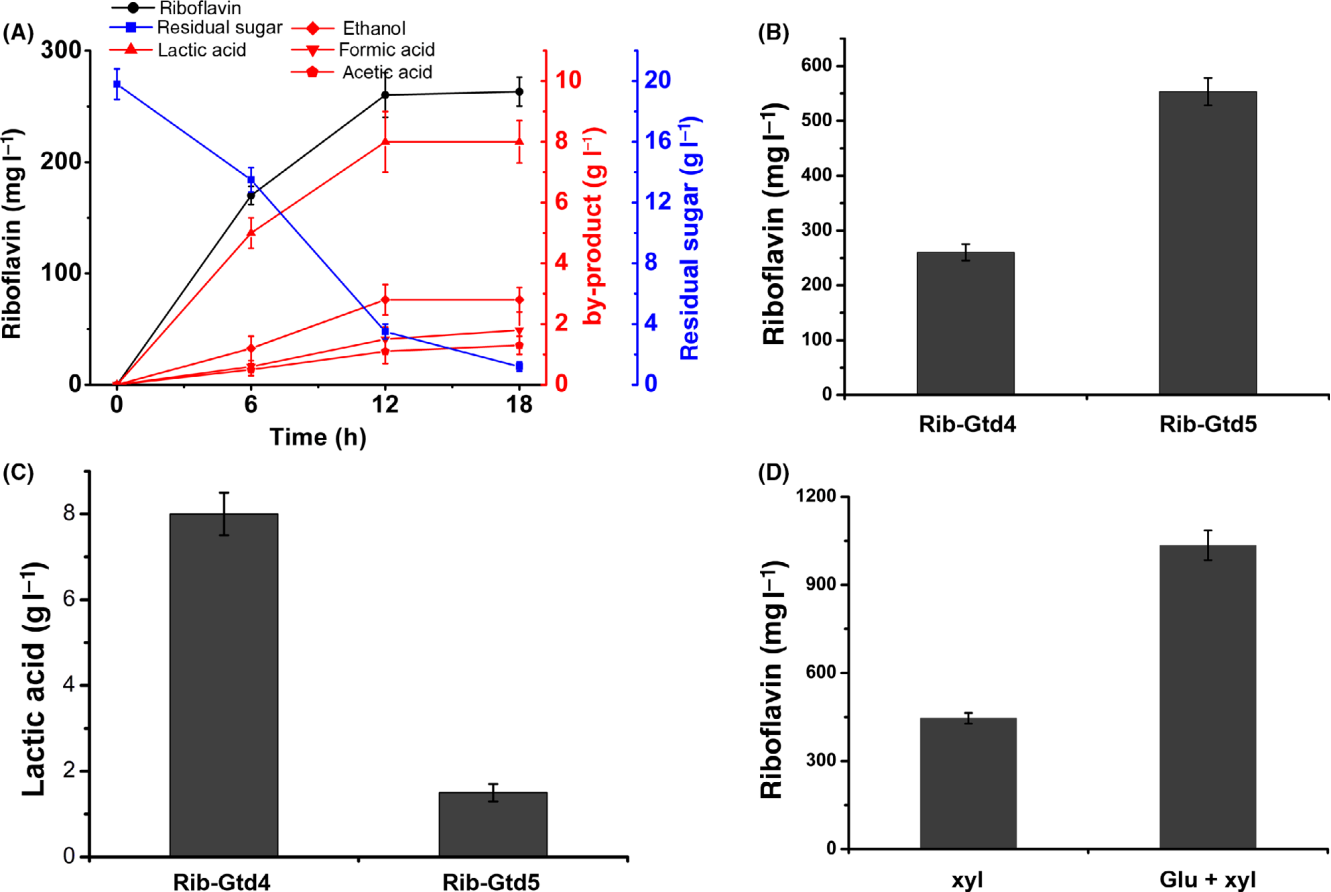
Elimination of main competing pathways in carbon metabolism. A. Production of metabolites by engineered strains of *G. thermoglucosidasius* Rib‐Gtd4. B. Elimination of *ldh*_Gtg_ boosted riboflavin production. C. Elimination of *ldh*_Gtg_ reduced lactic acid production. D. Riboflavin titres of the engineered strain of Rib‐Gtd5 in xylose and mixtures of glucose–xylose mineral media. Data shown are the average and SD of three independent experimental replicates.

It was reported that the wild‐type strains displayed a typical mixed‐acid fermentation with glucose as substrate. We therefore characterized the product composition of the finally engineered strain. As shown in Figure 6A, the main product of Rib‐Gtd4 was still lactic acid, together with relatively low amounts of ethanol and acetic acids. Thus, we deleted the lactic dehydrogenase gene (AOT13_05975, *ldh*_Gtg_) in Rib‐Gtd4, resulting in strain Rib‐Gtd5 (Fig. S11). As expected, we observed the significant improvement of riboflavin titre, reaching 553.3 ± 25 mg l^−1^ from 260.3 ± 15 mg l^−1^ (Fig. 6B). We further measured the production level of lactic acid and found it still presented in the fermentation broth of Rib‐Gtd5 (Fig. 6C), indicating the presence of a second lactate dehydrogenase gene in the organism or of an alternative pathway for lactate formation.

*G. thermoglucosidasius* is well known to ferment a wide range of sugars, such as glucose, cellobiose, xylose and mixtures of glucose, xylose and arabinose. We next tested the riboflavin titres of the engineered strain of Rib‐Gtd5 in mineral salt medium with xylose or mixtures of glucose–xylose. As shown in Figure 6D, we obtained 445.8 mg l^−1^ riboflavin after 12 h fermentation when using xylose as main carbon source, which is a little less than that of using glucose as main carbon source. However, when using the mixtures of glucose–xylose as carbon source, we observed a much higher titre of 1034.5 mg l^−1^ riboflavin (Fig. 6D), indicating *G. thermoglucosidasius* Rib‐Gtd5 is a promising thermophilic host to produce riboflavin.

## Discussion

For sustainable development in biochemical processes, efforts have been directed towards process intensification. Through process intensification, biotechnology companies try to enhance production of biological products by reducing energy consumption (Piemonte *et al.*, 2013; Gao *et al.*, 2017). In terms of energy consumption, using thermo‐tolerant microorganisms is one of the possible approaches for delivering more economical bio‐products (Abdel‐Banat *et al.*, 2010; Bhatia *et al.*, 2012). Therefore, fermentation at higher temperatures (above 37°C) (Abdel‐Banat *et al.*, 2010) has received much attention due to increase in the rate of production, reduction in cooling costs and decrease in the risk of contamination (Abdel‐Banat *et al.*, 2010; Eiadpum *et al.*, 2012; Shafiei *et al.*, 2017). In this work, we demonstrated that *G thermoglucosidasius* DSM2542 is a promising host to develop high‐temperature cell factory for riboflavin production. Since riboflavin fermentation is an exothermic process, it seems that using a thermo‐tolerant microorganism may help to reduce the cost of production.

Species in the *Geobacillus* genus are capable of growth between 40°C and 70°C. Among them, *G. thermoglucosidasius* tends to ferment a wide range of substrates, utilizing both cellobiose and pentose sugars at 60°C (Nazina *et al.*, 2001). This strain was successfully used for the production of ethanol and isobutanol (Cripps *et al.*, 2009; Lin *et al.*, 2014; Zhou *et al.*, 2016). Both products are derived from glycolysis pathway. Due to the characteristic of fast growth at diverse substrates, we speculated that other necessary pathway for growth (such as PP pathway) might also be used for economical bio‐production. The precursors for riboflavin biosynthesis all originate from PP pathway; moreover, the current mesophilic fermentation of riboflavin needs considerable energy cost for fermenter cooling (Baltz *et al.*, 2010). Therefore, as proof of concept, we engineered the thermophilic host *G thermoglucosidasius* DSM2542 to produce riboflavin. After a series of engineering, our endeavour indicated that, as an ideal thermophilic host for riboflavin production, *G. thermoglucosidasius* exhibited great potential for further development. Also, this work will provide reference for the application of *G. thermoglucosidasius* to produce other value‐added chemicals derived from PP pathway.

Commercial riboflavin production has been achieved using a combination of classical mutagenesis, genome shuffling and rational metabolic engineering in *B. subtilis* (Perkins *et al.*, 1999; Wang *et al.*, 2011, 2011, 2014; Revuelta, *et al.*, 2016; Schwechheimer, *et al.*, 2016). In addition, Wang *et al.* (2018) have systematically analysed the relationship between the genetic characteristics and riboflavin overproduction phenotype of *B. subtilis*. Definitely, these identified beneficial mutations can then be further combined into the thermophilic strain *G. thermoglucosidasius* to generate an alternative energy‐saving producer. Especially, we had developed a very convenient method for gene ‘knock‐out’ and ‘knock‐in’ in this thermophilic host, which avoided an extensive screening process to isolate the clones that the integrated plasmid occurred the second‐round cross‐over. Although we do not further improve the engineered strain and test the performance in bioreactor, as proof of concept, our work demonstrated the feasibility of riboflavin production by engineering the thermophilic *G. thermoglucosidasius*. More importantly, the present work paves the way to further engineer this promising host for riboflavin production.

## Experimental procedures

### Strains and culture conditions

Bacterial strains used in this work are listed in Table S1. *G. thermoglucosidasius* DSM 2542 and *G. thermodenitrificans* NG80‐2 were purchased from China General Microbiological Culture Collection Center (CGMCC). *Escherichia coli* JM109 were genetically manipulated according to the standard protocols (Green and Sambrook, 2012). LB medium was used to cultivate *E. coli* JM109 and *B. subtilis* (Liang *et al.*, 2019). Cultures (50 ml) were routinely grown in 250 ml flasks at 220 rpm and 37°C. For fermentation of *G. thermoglucosidasius*, modified ASYE mineral salt medium was used (20 g l^−1^ glucose, 5 g l^−1^ yeast extract, 6.78 g l^−1^ Na_2_HPO_4_, 3 g l^−1^ KH_2_PO_4_, 1 g l^−1^ NH_4_Cl, 0.986 g l^−1^ MgSO_4_·7H_2_O, 0.5 g l^−1^ NaCl, 0.42 g l^−1^ citric acid, 0.028 g l^−1^ FeSO_4_·7H_2_O, 0.022 g l^−1^ CaCl_2_, 0.01 g l^−1^ thiamin, Trace Metal Mix (4.4 mg l^−1^ NiSO_4_·6H_2_O, 2.86 mg l^−1^ H_3_BO_3_, 1.81 mg l^−1^ MnCl_2_·4H_2_O, 0.39 mg l^−1^ Na_2_MoO_4_·2H_2_O, 0.222 mg l^−1^ ZnSO_4_·7H_2_O, 0.079 mg l^−1^ CuSO_4_·5H_2_O, 0.049 mg l^−1^ Co(NO_3_)_2_·6H_2_O), 3.1 mg l^−1^ biotin and 47.66 g l^−1^ HEPES buffer; Lin *et al.*, 2014). When required, antibiotics were added to the growth media at the following concentrations: 100 μg ml^−1^ of ampicillin for *E. coli* and 12.5 μg ml^−1^ kanamycin for *G. thermoglucosidasius*.

### Chemicals and reagents

All chemicals were acquired from Sigma‐Aldrich or Thermo Scientific. Phusion High‐Fidelity DNA Polymerase, T4 polymerase and restriction enzymes were purchased from New England Biolabs.

### Construction of plasmids

To express native and heterogeneous rib cluster in *G. thermoglucosidasius* DSM 2542, the rib clusters were cloned from *Bacillus subtilis* 168, *G. thermodenitrificans* NG80‐2 and the native host using primer pair 168rib‐F/168rib‐R, NG80rib‐F/NG80rib‐R and DSM2542rib‐F/DSM2542rib‐R respectively. The promoter used for driving these *rib* clusters was amplified from genomic DNA of *G. thermoglucosidasius* NG80‐2. Then, Gibson assembled (Gibson *et al.*, 2009) with plasmid backbone amplified from pucG3.8 to generate plasmid pUCG‐Bs, pUCG‐Gtd and pUCG‐Gtg.

To test stability of the visible markers in *G. thermoglucosidasius* DSM 2542, the *ldh* promoter was amplified by PCR from genomic DNA of *G. thermoglucosidasius* NG80‐2 using primer pair Pldh‐F/Pldh‐R. *sfgfp* was amplified by PCR from plasmid pTAC‐RiboJ‐gfp (Lou *et al.*, 2012) using primer pair sfGFP‐F/sfGFP‐R. Subsequently, the two DNA fragments and the plasmid backbone amplified from pUB31 using primer pair Vs‐F/Vs‐R were assembled using Gibson Assembly to generate plasmid pUB‐sfGFP. Similarly, *idgS‐sfp* and *xylE* genes were amplified from pCIM002 (Xie *et al.*, 2017) and pCSW3 (Wang *et al.*, 2015) and assembled with *ldh* promoter and plasmid backbone of pUB31 to generate plasmid pUB‐idgS‐sfp and pUB‐xylE.

For gene in‐frame deletion, briefly, the upstream and downstream fragments of the coding sequence of a *pyrE*_Gtg,_ *purR*_Gtg_, *purA*_Gtg_, *ccpN*_Gtg_ and *ldh*_Gtg_ were amplified by PCR. Then, plasmid backbone amplified from pUB‐sfGFP using primer pair pUB31s/pUB31a and DNA fragments were assembled with Gibson Assembly to generate plasmid pUB‐pyrE, pUB‐purR, pUB‐purA, pUB‐ccpN and pUB‐ldh respectively. For *ribC*_Gtg_ gene point mutation, the main procedure of plasmid construction was essentially the same as that of gene deletion expect that the *ribC*_Gtg_ gene point mutation was introduced by primers. Plasmids pUB‐ribC was constructed for the point mutation of RibC_Gtg_ in *G. thermoglucosidasius* DSM2542.

### Transformation of *G. thermoglucosidasius*

*G. thermoglucosidasius* DSM 2542 transformation was conducted as described previously (Cripps, *et al.*, 2009).

### Measurement of biomass and riboflavin

Cell growth was monitored by measuring optical density at 600 nm (OD_600_) with UV–Vis spectrophotometer. For riboflavin measurement, samples were first diluted with 0.05 M NaOH and centrifuged at 16 000 × *g* for 2 min to remove the cells, the samples were then subjected to HPLC analysis using a Agilent 1260 system equipped with a YMC polymer C18 Column (4.6 × 250 mm) and riboflavin was detected by UV 370 nm (Wang *et al.*, 2019). Separation was performed at the following conditions: 60% H_2_O, 10% methanol, 20% acetonitrile and 10% phosphoric acid (2 mM) with a constant flow rate of 1 ml min^−1^.

### DNA replacement

Integration vectors for gene replacement were introduced into *G. thermoglucosidasius* by electroporation, and transformants were selected on TSA (Sheng *et al.*, 2017) agar containing 12.5 mg ml^−1^ kanamycin at 52°C. Integration by homologous recombination at the genomic allele was forced by growing transformants in shaken liquid cultures (2TY, 8–16 h at 60°C) and plating on the same solid medium at 68°C for 24 h. At this temperature, the integration vectors are unable to replicate autonomously and a high percentage of the colonies arising had the desired mutant phenotype.

Clones of stable integrants were then cultured without kanamycin in liquid TGP media at 60°C for 6–8 h. Subsequently, presumptive double cross‐overs were screened on TSA agar without kanamycin and green fluorescence. The double cross‐over formation was confirmed by PCR, using genomic DNA and primers listed in Table S2.

### Determination the relative activity of mutant RibC_Gtg_

Cell extracts preparation of GT‐01 and *G. thermoglucosidasius* DSM2542 strains. The enzyme activity and HPLC analysis of two strains were prepared as previously described (Mack *et al.*, 1998), except that the reaction temperature here is 55°C.

### Analysis of gene expression by quantitative RT‐PCR

Cells were harvested in mid‐exponential phase and immediately transferred into liquid nitrogen to block the metabolism. Total RNA was isolated using RNAprep Pure Cell/Bacteria Kit (Tiangen Biotech, Beijing, China) according to the manufacture’s recommendation, and the RNA quantitation was performed at 260/280 nm using a NanoDrop ND‐2000 spectrophotometer (Thermo Scientific, Wilmington, DE, USA).

For real‐time RT‐qPCR experiments, the first‐strand synthesis of cDNA was carried out using 1 μg total RNA with a PrimeScript™ RT Reagent Kit with gDNA Eraser (TaKaRa, Japan) following the manufacturer’s instructions. Oligonucleotides used for RT‐qPCR are listed in Table S2.

The quantitative RT‐PCR procedures were performed as previously described (Cao *et al.*, 2018). Results were collected and analysed using the supporting 7500 software (v2.0.4). Based on the 2^−ΔΔ^ *^Ct^* method (Livak and Schmittgen, 2001), we calculated the gene expression level using housekeeping gene *gap* as reference gene (Song *et al.*, 2016). Technical triplicates of three biological repeats were performed per condition.

### Analysis of fermentation products

Organic acid and ethanol were quantified in clarified culture samples by HPLC using an Agilent 1260 system equipped with an Aminex HPX‐87H Ion Exclusion Column (Bio‐Rad Laboratories, Richmond, CA, USA) and refractive index detector. Organic acids were detected by UV 215 nm, and ethanol was detected by refractive index detector. The column was eluted with 5 mM sulphuric acid at a flow rate of 1 ml min^−1^. The measuring method of riboflavin and glucose procedures was performed as previously described (Shi *et al.*, 2014).

## Conflict of interest

The authors declare no conflict of interest. We have filed a provisional patent for the present work.

## Supporting information


**Figure S1.** Growth curves of *G. thermoglucosidasius* DSM2542 in 2SPY liquid medium.
**Figure S2.** Titers of *G. thermoglucosidasius* DSM2542 with native or heterogeneous *rib* clusters. Rib‐C is *G. thermoglucosidasius* DSM2542 with pUCG3.8 plasmid. Rib‐Bs, Rib‐Gtg and Rib‐Gtd are *G. thermoglucosidasius* DSM2542 with the *rib* from *Bacillus subtilis* 168, *G. thermoglucosidasius* DSM2542 and *G. thermodenitrificans* NG80‐2, respectively.
**Figure S3.** Selection of the second‐round double cross‐over using our developed DNA replacement method. (A) Second‐round double cross‐over clones could be observed by the phenotype of fluorescence loss. Here *pyrE* was chosen as target to delete. (B) Confirmation of the desired knockout mutant by PCR. wild‐type, 1205 bp; knockout mutant, 700 bp. 12 colonies without green fluorescence acquired from the left plate were identified by PCR using primer pairs pyrE‐F/pyrE‐R (Table S2). *G. thermoglucosidasius* DSM2542 genomic DNA was used as a negative control (‐). Plasmid pUB‐purE for knockout was used as a positive control (+). Lane M, DNA size marker.
**Figure S4.** Alignment of RibC and RibCGtg in *B. subtilis* 168 and *G. thermoglucosidasius* DSM2542, respectively. Red letter marked triangle is the mutant site. Here we mutated the G199 to D.
**Figure S5.** Confirmation of point mutation in *ribC*_Gtg_(G199D) by sequencing.
**Figure S6.** Alignment of PurR and PurR_Gtg_ in *B. subtilis* 168 and *G. thermoglucosidasius* DSM2542, respectively.
**Figure S7.** Construction of purR_Gtg_ knockout strain. (A) Schematic of genetic information for wild‐type (WT) and knockout (KO) strains. LF and RF indicate the sequences upstream and downstream of purR_Gtg_ used for knockout. The arrows indicate primer pairs purR_Gtg_‐F/purR_Gtg_‐R used for PCR confirmation. (B) Confirmation of the desired knockout mutant by PCR. wild‐type, 1013 bp; knockout mutant, 509 bp.
**Figure S8.** Construction of purR_Gtg_ knockout strain. (A) Schematic of genetic information for wild‐type (WT) and knockout (KO) strains. LF and RF indicate the sequences upstream and downstream of purR_Gtg_ used for knockout. The arrows indicate primer pairs purR_Gtg_‐F/purR_Gtg_‐R used for PCR confirmation. (B) Confirmation of the desired knockout mutant by PCR. wild‐type, 1013 bp; knockout mutant, 509 bp.
**Figure S9.** Alignment of CcpN and CcpN_Gtg_ in *B. subtilis* 168 and *G. thermoglucosidasius* DSM2542, respectively.
**Figure S10.** Construction of ccpN_Gtg_ knockout strain. (A) Schematic of genetic information for wild‐type (WT) and knockout (KO) strains. LF and RF indicate the sequences upstream and downstream of ccpN_Gtg_ used for knockout. The arrows indicate primer pairs ccpN_Gtg_‐F/ccpN_Gtg_‐R used for PCR confirmation. (B) Confirmation of the desired knockout mutant by PCR. wild‐type, 895 bp; knockout mutant, 437 bp.
**Figure S11.** Construction of ccpN_Gtg_ knockout strain. (A) Schematic of genetic information for wild‐type (WT) and knockout (KO) strains. LF and RF indicate the sequences upstream and downstream of ccpN_Gtg_ used for knockout. The arrows indicate primer pairs ccpN_Gtg_‐F/ccpN_Gtg_‐R used for PCR confirmation. (B) Confirmation of the desired knockout mutant by PCR. wild‐type, 895 bp; knockout mutant, 437 bp.
**Table S1.** The strains and plasmids used and constructed in this study.
**Table S2.** Primers used in this study.
