# Supplementary material for: Engineering thermophilic Geobacillus thermoglucosidasius for riboflavin production
Source: Microb Biotechnol. 2020 Feb 25;14(2):363–73. doi: 10.1111/1751-7915.13543 (PMC7936320; doi:10.1111/1751-7915.13543)
Supplement: Supplementary file 1 — Figure S1. Growth curves of G. thermoglucosidasius DSM2542 in 2SPY liquid medium. Figure S2. Titers of G. thermoglucosidasius DSM2542 with native or heterogeneous rib clusters. Rib‐C is G. thermoglucosidasius DSM2542 with pUCG3.8 plasmid. Rib‐Bs, Rib‐Gtg and Rib‐Gtd are G. thermoglucosidasius DSM2542 with the rib from Bacillus subtilis 168, G. thermoglucosidasius DSM2542 and G. thermodenitrificans NG80‐2, respectively. Figure S3. Selection of the second‐round double cross‐over using our developed DNA replacement method. (A) Second‐round double cross‐over clones could be observed by the phenotype of fluorescence loss. Here pyrE was chosen as target to delete. (B) Confirmation of the desired knockout mutant by PCR. wild‐type, 1205 bp; knockout mutant, 700 bp. 12 colonies without green fluorescence acquired from the left plate were identified by PCR using primer pairs pyrE‐F/pyrE‐R (Table S2). G. thermoglucosidasius DSM2542 genomic DNA was used as a negative control (‐). Plasmid pUB‐purE for knockout was used as a positive control (+). Lane M, DNA size marker. Figure S4. Alignment of RibC and RibCGtg in B. subtilis 168 and G. thermoglucosidasius DSM2542, respectively. Red letter marked triangle is the mutant site. Here we mutated the G199 to D. Figure S5. Confirmation of point mutation in ribC Gtg(G199D) by sequencing. Figure S6. Alignment of PurR and PurRGtg in B. subtilis 168 and G. thermoglucosidasius DSM2542, respectively. Figure S7. Construction of purRGtg knockout strain. (A) Schematic of genetic information for wild‐type (WT) and knockout (KO) strains. LF and RF indicate the sequences upstream and downstream of purRGtg used for knockout. The arrows indicate primer pairs purRGtg‐F/purRGtg‐R used for PCR confirmation. (B) Confirmation of the desired knockout mutant by PCR. wild‐type, 1013 bp; knockout mutant, 509 bp. Figure S8. Construction of purRGtg knockout strain. (A) Schematic of genetic information for wild‐type (WT) and knockout (KO) strains. LF and RF indi [file MBT2-14-363-s001.pdf]

Engineering thermophilic *Geobacillus thermoglucosidasius* for riboflavin production

Zhiheng Yang[1,#], Qingqing Sun[2,#], Gaoyi Tan[1], Quanwei Zhang[2], Zhengduo Wang[1], Chuan Li[1], Fengxian Qi[2], Weishan Wang[1,2,*], Lixin Zhang[1,*] and Zilong Li[2,*]

[1]*State Key Laboratory of Bioreactor Engineering, East China University of Science and Technology, Xuhui District, Shanghai 200237, China*

[2]*State Key Laboratory of Microbial Resources, Institute of Microbiology, Chinese Academy of Sciences, Chaoyang District, Beijing 100101, China*

[#] These authors contributed equally to this work

[*] For correspondence. E-mail: wangws@im.ac.cn, lizl@im.ac.cn, and lxzhang@ecust.edu.cn; Tel./Fax +86-021-64252575.

Contents

Fig. S1 Growth curves of *G. thermoglucosidasius* DSM2542 in 2SPY liquid medium. 3

Fig. S2 Titers of *G. thermoglucosidasius* with native or heterogeneous *rib* clusters 4

Fig. S3 Selection of the second-round double cross-over 5

Fig. S4 Alignment of RibC and RibC$_{\mathrm{Gtg}}$ 6

Fig. S5 Confirmation of point mutation in *ribC*$_{\mathrm{Gtg}}$(G199D) by sequencing................................. 7

Fig. S6 Alignment of PurR and PurR$_{\mathrm{Gtg}}$... 8

Fig. S7 Construction of *purR*$_{\mathrm{Gtg}}$ knockout strain. 9

Fig. S8 Construction of *purA*$_{\mathrm{Gtg}}$ knockout strain. 10

Fig. S9 Alignment of CcpN and CcpN$_{\mathrm{Gtg}}$ 11

Fig. S10 Construction of *ccpN*$_{\mathrm{Gtg}}$ knockout strain. 12

Fig. S11 Construction of *ldh*$_{\mathrm{Gtg}}$ knockout strain. 13

Table S1 The strains and plasmids used and constructed in this study 14

Table S2 Primers used in this study 16

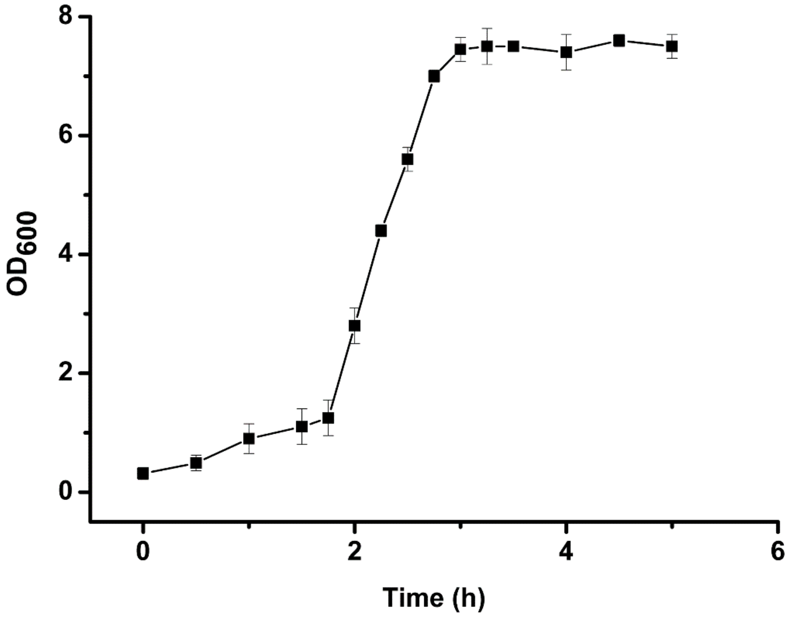

Fig. S1 Growth curves of *G. thermoglucosidasius* DSM2542 in 2SPY liquid medium.

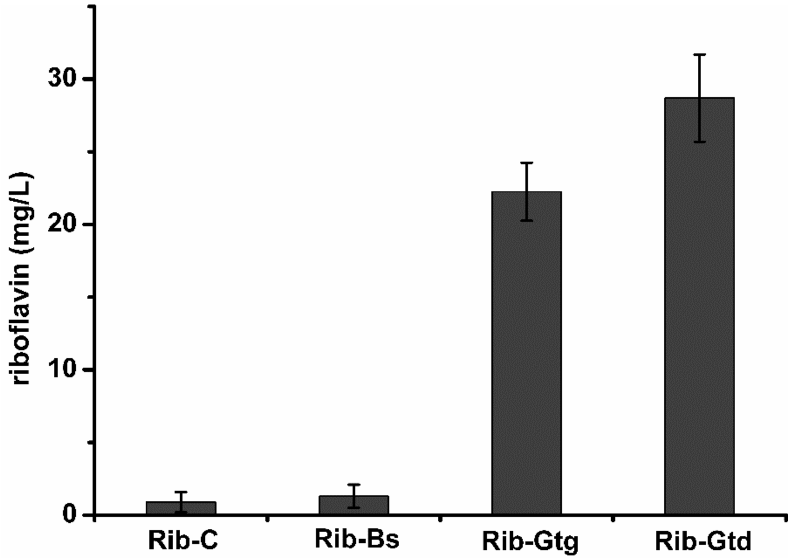

Fig. S2 Titers of *G. thermoglucosidasius* DSM2542 with native or heterogeneous *rib* clusters. Rib-C is *G. thermoglucosidasius* DSM2542 with pUCG3.8 plasmid. Rib-Bs, Rib-Gtg and Rib-Gtd are *G. thermoglucosidasius* DSM2542 with the *rib* from *Bacillus subtilis* 168, *G. thermoglucosidasius* DSM2542 and *G. thermodenitrificans* NG80-2, respectively.

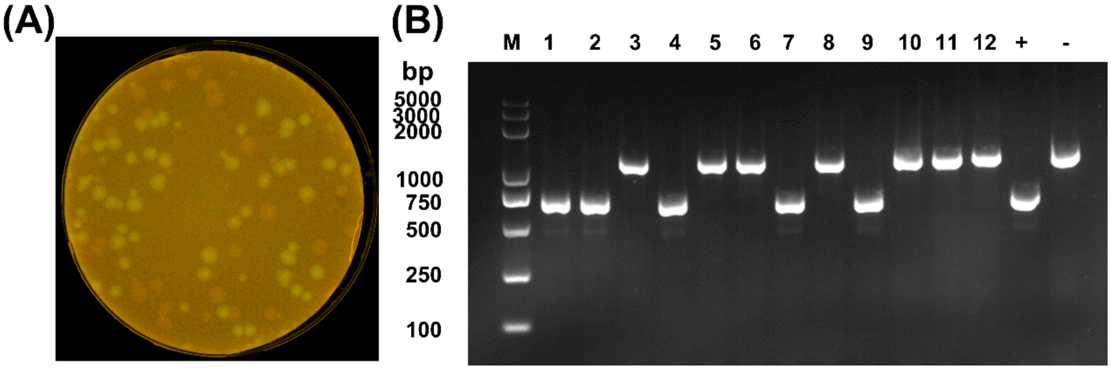

Fig. S3 Selection of the second-round double cross-over using our developed DNA replacement method.

(A) Second-round double cross-over clones could be observed by the phenotype of fluorescence loss. Here *pyrE* was chosen as target to delete. (B) Confirmation of the desired knockout mutant by PCR. wild-type, 1205 bp; knockout mutant, 700 bp. 12 colonies without green fluorescence acquired from the left plate were identified by PCR using primer pairs pyrE-F/pyrE-R (Table S2). G. thermoglucosidasius DSM2542 genomic DNA was used as a negative control (-). Plasmid pUB-purE for knockout was used as a positive control (+). Lane M, DNA size marker.

```
RibC        MKTIHITHPHHLIKEEQAKSVMALGYFDGVHLGHQKVIGTAKQIAEEKGLTLAVMTFHPH
RibCGtg     MKTLFISHPHQMKKEELPPTVMALGYFDGIHLGHQKVIRTAVQIAAEKGYKSAVMTFHPH
            ***:.*:***:: *** .  :*********:******** ** *** *** .  ********

RibC        PSHVLGRDKEPKDLITPLEDKINQIEQLGTEVLYVVKFNEVFASLSPKQFIDQYIIGLNV
RibCGtg     PSVVLGKKDKHVHLITPLKKKEQLIGELGIDYLYIVEFTSSFAQLFPQEFVDQYIIGLHV
            ** ***:..:  .*****:.* : * :** : **:*:*..  **.* *::*:*******:*

RibC        QHAVAGFDFTYGKYGKGTMKTMPDDLDGKAGCTMVEKLTEQDKKISSSYIRTALQNGDVE
RibCGtg     KHVVAGFDFTYGRLGKGTMETLPFHSREQFTQTVIPKLSIDGEKISSTYVRQLLKNGDVD
            :*.*********: *****:*:* .    :   *:: **: :.:****:*:*  *:****:
                            ▼
RibC        LANVLLGQPYFIKGIVIHGDKRGRTIGFPTANVGLNNSYIVPPTGVYAVKAEVNGEVYNG
RibCGtg     QLPRLLGRFYEVEGTVVGGERRGRTIGFPTANIALKDDYLLPALGVYAVKVKIGSDIFEG
                ***: * ::* *: *::***********:.*::.*::*. ******.::..::::*

RibC        VCNIGYKPTFYEKRPEQPSIEVNLFDFNQEVYGAAIKIEWYKRIRSERKFNGIKELTEQI
RibCGtg     VCNVGYKPTFYSTREGLPSIEVHIFDFAKDIYGETMTIEWHMRLRSEQKFAGVDELIAQI
            ***:*******..*   *****::*** :::** ::.***: *:***:** *:.**  **

RibC        EKDKQEAIRYFSNLRK-----------
RibCGtg     QRDKEKAQAYFRNFAETTCILSQKEVF
            ::**::*  ** *: :
```

Fig. S4 Alignment of RibC and $RibC_{Gtg}$ in *B. subtilis* 168 and *G. thermoglucosidasius* DSM2542, respectively. Red letter marked triangle is the mutant site. Here we mutated the G199 to D.

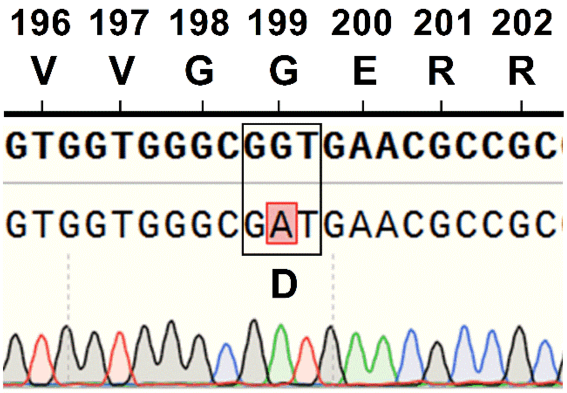

Fig. S5 Confirmation of point mutation in *ribC*$_{\mathrm{Gtg}}$(G199D) by sequencing

```
PurR       MKFRRSGRLVDLTNYLLTHPHELIPLTFFSERYESAKSSISEDLTIIKQTFEQQGIGTLL
PurRGtg    MKLRRSGRLVDMTHYLLERPHQLIPLTFFAERYESAKSSISEDLAIIKQTFEQQGIGTIK
           **:********:*:*** :**:*******:**************:*************:

PurR       TVPGAAGGVKYIPKMKQAEAEEFVQTLGQSLANPERILPGGYVYLTDILGKPSVLSKVGK
PurRGtg    TLPGAAGGVQYIPKMSRQEADGIVTYLCEQLSRPDRLLPGGYLYMTDILGDPRVVNKIGR
           *:*******:*****.: **: :*  * :.*:.*:*:*****:*:*****.* *:.*:*:

PurR       LFASVFAEREIDVVMTVATKGIPLAYAAASYLNVPVVIVRKDNKVTEGSTVSINYVSGSS
PurRGtg    LYASIFADRPVDVVMTIATKGIPLAYAVAHFLYVPVVIVRHDNKVTEGSMVSINYVSGSS
           *:**:**:* :*****:**********.* :* *******:******** **********

PurR       NRIQTMSLAKRSMKTGSNVLIIDDFMKAGGTINGMINLLDEFNANVAGIGVLVEAEGVDE
PurRGtg    KRIQTMVLAKRSLAEGANVLIIDDFMKAGGTVNGMINLLNEFNAKLSGIGVLVESEETKE
           :***** *****:  *:**************:*******:****:::*******:* ..*

PurR       RLVDEYMSLLTLSTINMKEKSIEIQNGNFLRFFKDNLLKNGETES
PurRGtg    RLVDEYISLVKLSSVDVKEKQITVKAGNYIHFME-----------
           ******:**:.**::::***.* :: **:::*::
```

Fig. S6 Alignment of PurR and PurR$_{Gtg}$ in *B. subtilis* 168 and *G. thermoglucosidasius* DSM2542, respectively.

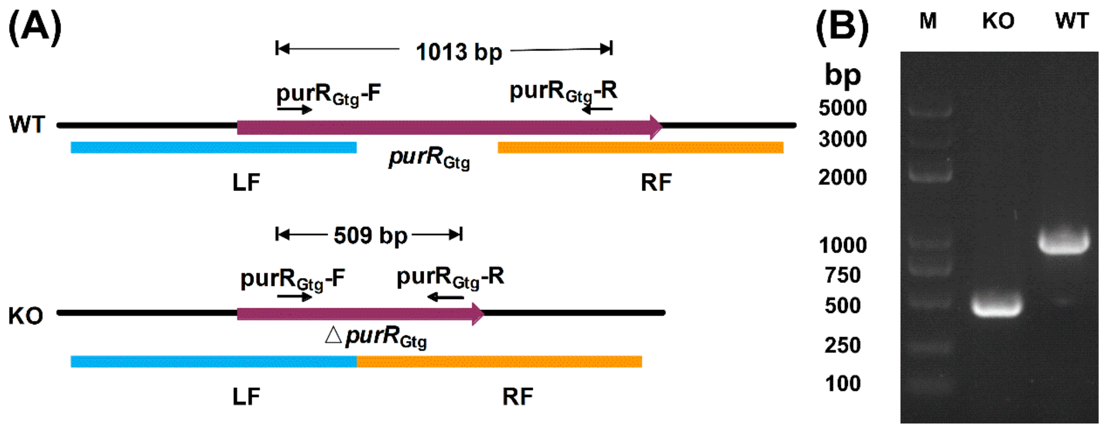

Fig. S7 Construction of *purR*$_{Gtg}$ knockout strain.

(A) Schematic of genetic information for wild-type (WT) and knockout (KO) strains. LF and RF indicate the sequences upstream and downstream of *purR*$_{Gtg}$ used for knockout. The arrows indicate primer pairs purR$_{Gtg}$-F/purR$_{Gtg}$-R used for PCR confirmation. (B) Confirmation of the desired knockout mutant by PCR. wild-type, 1013 bp; knockout mutant, 509 bp.

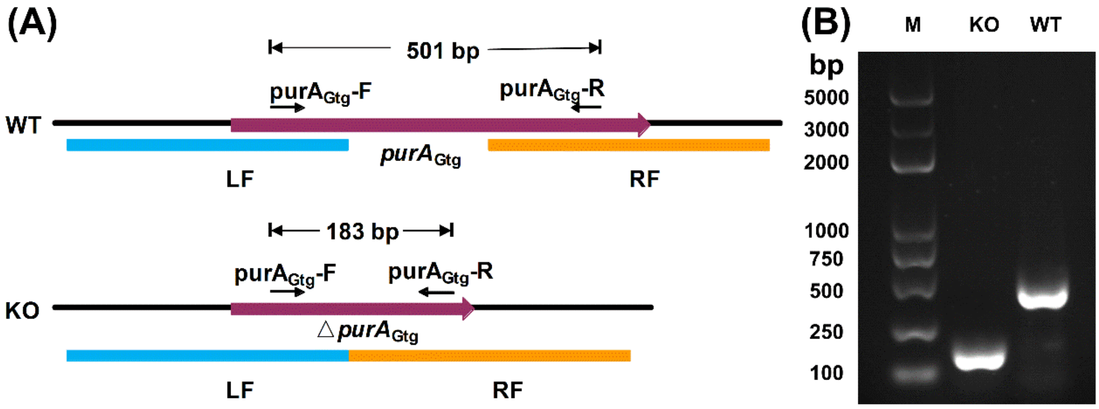

Fig. S8 Construction of *purA*$_{\mathrm{Gtg}}$ knockout strain.

(A) Schematic of genetic information for wild-type (WT) and knockout (KO) strains. LF and RF indicate the sequences upstream and downstream of *purA*$_{\mathrm{Gtg}}$ used for knockout. The arrows indicate primer pairs purA$_{\mathrm{Gtg}}$-F/purA$_{\mathrm{Gtg}}$-R used for PCR confirmation. (B) Confirmation of the desired knockout mutant by PCR. wild-type, 501 bp; knockout mutant, 183 bp**.**

```
CcpN          MSTIELNKRQEHILQIVKENGPITGEHIAEKLNLTRATLRPDLAILTMSGFLEARPRVGY
CcpNGtg       -------------MQIVKDHGPITGESIAEKLNLTRATLRPDLAILTMAGYLEARPRVGY
                           :****::****** *********************:*:*********

CcpN          FYTGKTGTQLLADKLKKLQVKDFQSIPVVIHENVSVYDAICTMFLEDVGTLFVVDRDAVL
CcpNGtg       FYTGKTGSQLLADKIKKIKVEDYQSIPVVVNENVSVYDAIVTMFLEDVGTLFVVDDEALL
              *******:******:**::*:*:******::********* ************** :*:*

CcpN          VGVLSRKDLLRASIGQQELTSVPVHIIMTRMPNITVCRREDYVMDIAKHLIEKQIDALPV
CcpNGtg       AGVLSRKDLLRASIGKQELTTIPVNIIMTRMPNVAVCYKDDPLIEVAERLIEKQIDAMPV
              .**************:****::**:********::** ::* ::::*::********:**

CcpN          IKDTDKGFEVIGRVTKTNMTKILVSLSENEIL
CcpNGtg       VRKTEKGYEVIGRITKTNMTKAFVALAKDDLL
              ::.*:**:*****:******* :*:*:::::
```

Fig. S9 Alignment of CcpN and $CcpN_{Gtg}$ in *B. subtilis* 168 and *G. thermoglucosidasius* DSM2542, respectively.

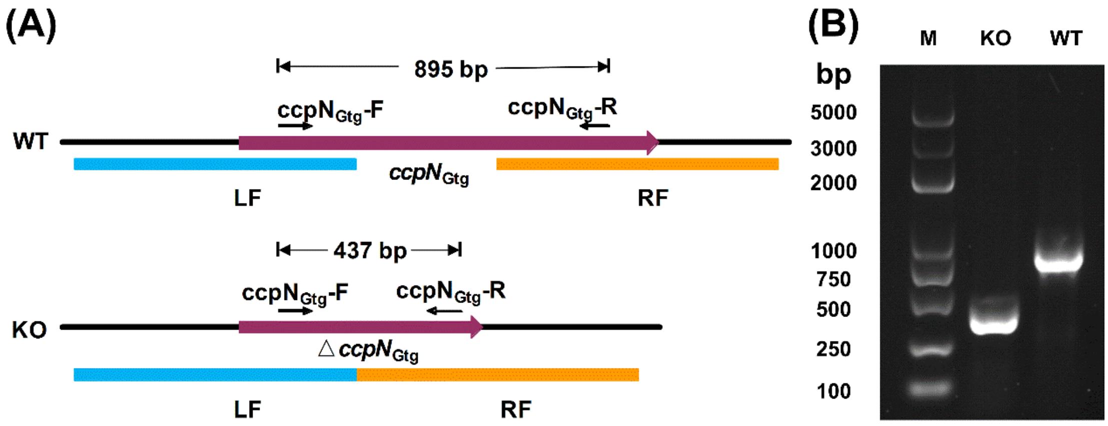

Fig. S10 Construction of *ccpN*Gtg knockout strain.

(A) Schematic of genetic information for wild-type (WT) and knockout (KO) strains. LF and RF indicate the sequences upstream and downstream of *ccpN*Gtg used for knockout. The arrows indicate primer pairs ccpNGtg-F/ccpNGtg-R used for PCR confirmation. (B) Confirmation of the desired knockout mutant by PCR. wild-type, 895 bp; knockout mutant, 437 bp.

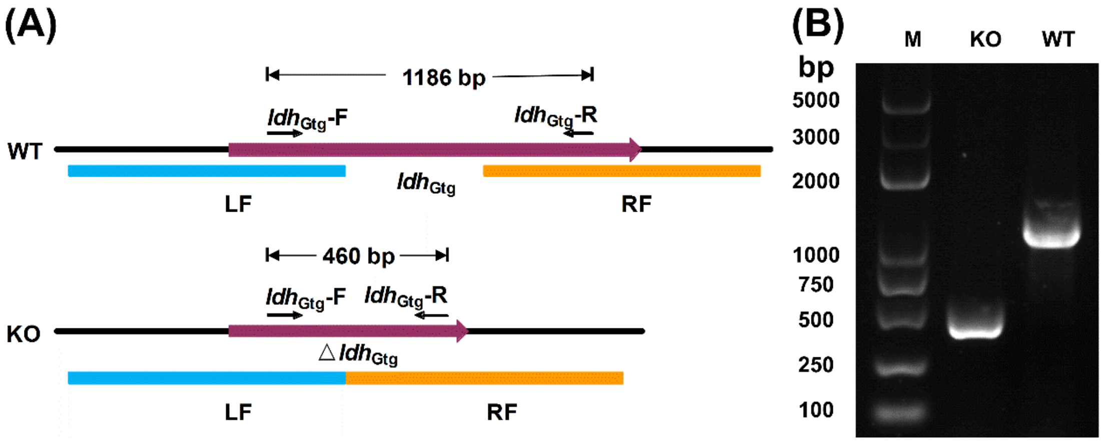

Fig. S11 Construction of *ldh*Gtg knockout strain.

(A) Schematic of genetic information for wild-type (WT) and knockout (KO) strains. LF and RF indicate the sequences upstream and downstream of *ldh*Gtg used for knockout. The arrows indicate primer pairs ldhGtg-F/ldhGtg-R used for PCR confirmation. (B) Confirmation of the desired knockout mutant by PCR. wild-type, 1186 bp; knockout mutant, 460 bp.

Table S1 The strains and plasmids used and constructed in this study

Stains	Description	Source
E. coli JM109	General cloning host for plasmid manipulation	Novagen
G. thermodenitrificans NG80-2	Wide-type strain	Lab stock
G.thermoglucosidasius DSM2542	Wide-type strain	Lab stock
B. subtilis 168	Wide-type strain	Lab stock
Rib-Gtd	*G. thermoglucosidasius* DSM2542 containing pUCG-Gtd	This study
GT-01	*G. thermoglucosidasius* DSM2542 with $RibC_{Gtg}$(G199D) allele mutant	This study
Rib-Gtd1	GT-01 containing pUCG-Gtd	This study
GT-02	*G. thermoglucosidasius* DSM2542 with $RibC_{Gtg}$(G199D) allele mutant, Δ*purA*	This study
Rib-Gtd2	GT-02 containing pUCG-Gtd	This study
GT-03	*G. thermoglucosidasius* DSM2542 with $RibC_{Gtg}$(G199D) allele mutant, Δ*purA*, Δ*purR*	This study
Rib-Gtd3	GT-03 containing pUCG-Gtd	This study
GT-04	*G. thermoglucosidasius* DSM2542 with $RibC_{Gtg}$(G199D) allele mutant, Δ*purA*, Δ*purR*, Δ*ccpN*	This study
Rib-Gtd4	GT-04 containing pUCG-Gtd	This study
GT-05	*G. thermoglucosidasius* DSM2542 with $RibC_{Gtg}$(G199D) allele mutant, Δ*purA*, Δ*purR*, Δ*ccpN*, Δ*ldh*	This study
Rib-Gtd5	GT-05 containing pUCG-Gtd	This study
plasmid	Description	Source
pUCG3.8	a minimal three-part *E. coli–Geobacillus* sp. shuttle vector	(Bartosiak-Jentys, et al., 2013)
pUCG-Gtd	Inserting rib_{Gtd} of *G. thermodenitrificans* NG80-2 into pUCG3.8	This study
pUCG-Gtg	Inserting rib_{Gtg} of *G. thermoglucosidasius* DSM2542 into pUCG3.8	This study
pUCG-Bs	Inserting rib_{Bs} of *B. subtilis* into pUCG3.8	This study
pUB31	The thermostable variant of kan^R from pUCG3.8	(Christopher A. Hills, 2015)
pTAC-RiboJ-gfp	Containing *sfgfp* gene	(Lou, et al.,

		2012)
pUB-sfGFP	*amp*^{R}; *kan*^{R}; *sfgfp*; *E. coli- G. thermoglucosidasius* DSM2542 shuttle vector	This study
pCIM002	Containing *idgs-sfp* gene	(Xie, et al., 2017)
pCSW3	Containing *xylE* gene	(Wang, et al., 2015)
pUB-idgS-sfp	*amp*^{R}; *kan*^{R}; *idgS-sfp*; *E. coli- G. thermoglucosidasius* DSM2542 shuttle vector	This study
pUB-xylE	*amp*^{R}; *kan*^{R}; *xylE*; *E. coli- G. thermoglucosidasius* DSM2542 shuttle vector	This study
pUB-pyrE	pUB-sfGFP containing *pyrE knock-out* cassette	This study
pUB-ribC	Inserting mutant *ribC* of *G. thermoglucosidasius* into pUB31	This study
pUB-purA	pUB-sfGFP containing *purA* knock-out cassette	This study
pUB-purR	pUB-sfGFP containing *purR* knock-out cassette	This study
pUB-ccpN	pUB-sfGFP containing *ccpN* knock-out cassette	This study
pUB-ldh	pUB-sfGFP containing truncated *ldh* knock-out cassette	This study

Bartosiak-Jentys J, Hussein AH, Lewis CJ, Leak DJ. (2013) Modular system for assessment of glycosyl hydrolase secretion in Geobacillus thermoglucosidasius. *Microbiology* **159**:1267-75.

Hills, C. (2015). Acetate metabolism in Geobacillus thermoglucosidasius and strain engineering for enhanced bioethanol production. [WWW document].URL https://researchportal.bath.ac.uk/en/studentTheses/acetate-metabolism-in-geobacillus-thermoglucosidasius-and-strain-

Lou C, Stanton B, Chen YJ, Munsky B, Voigt CA (2012) Ribozyme-based insulator parts buffer synthetic circuits from genetic context. *Nat Biotechnol* **30**:1137-42.

Xie, Z., Zhang, Z., Cao, Z., Chen, M., Li, P., Liu, W., et al. (2017) An external substrate-free blue/white screening system in Escherichia coli. *Appl Microbiol Biotechnol* **101**: 3811-3820.

Wang, W., Li, X., Li, Y., Li, S., Fan, K., and Yang, K. (2015) A genetic biosensor for identification of transcriptional repressors of target promoters. *Sci Rep* **5**: 15887.

Table S2 Primers used in this study

Name	Sequence
Pldh-F	ggagttaactgcctcgtcca
Pldh-R	cactattctcccttcttattattgtgaataca
sfGFP-F	tattcacaataataagaagggagaatagtgatgcgtaaaggcgaagagct
sfGFP-R	tgcaggtcgactctagaggatccgagtcactaagggctaacta
Vs-F	gtgactcggatcctctagagtcgacctgca
Vs-R	aaaaaaatggacgaggcagttaactccgaattcactggccgtcgttt
pyrE-F	ggtgcttcattatgcggcg
pyrE-R	tctctgtcatttcatcaccaatctt
idgS-sfp-F	ataataagaagggagaatagtgatgactcttcaggagaccag
idgS-sfp-R	tagaggatcttataaaagctcttcgtacgagac
Vi-F	tggtctcgtacgaagagcttttataagatcctctagagtcgacctgcag
Vi-R	gcaaaaaaatggacgaggcagttaactccgaattcactggccgtcgttt
xylE-F	aataataagaagggagaatagtgatgaaaaaaggagttatgcg
xylE-R	ggatctcaggtcagcacggtcatga
Vx-F	catgaccgtgctgacctgagatcctctagagtcgacctgcag
Vx-R	caaaaaaatggacgaggcagttaactccgaattcactggccgtcgttttac
NG80rib-F	gagcctttcgttttatttgatgcctggctcagctgcatgtttgacatttatcg
NG80rib-R	agggagaatagtgatgtacaatgatgaacattacatgc
NG80-F	cgttgtaaaacgacggccagtgaattcggagttaactgcctcgtcc
NG80-R	catcattgtacatcactattctcccttcttattattg
pucG3.8la	gaattcactggccgtcgttttacaacg
pucG3.8ls	gccaggcatcaaataaaacga
DSM2542rib-F	ctgagcctttcgttttatttgatgcctggcgtgcgaaacgatgaacaata
DSM2542rib-R	aagaagggagaatagtgttacaactcacatttatccaaaaatg
DSM2542-F	cgttgtaaaacgacggccagtgaattcggagttaactgcctcgtcc
DSM2542-R	atgtgagttgtaacactattctcccttcttattattg
168rib-F	gagcctttcgttttatttgatgcctggcttaattattgtatgaaatgtcttgatcctg
168rib-R	aagggagaatagtgatggaagagtattatatgaagctggc
168-F	cgttgtaaaacgacggccagtgaattcggagttaactgcctcgtcc
168-R	aatactcttccatcactattctcccttcttattattg
pUB31-F	tggcgtaatcatggtcatag
pUB31-R	cgagtcactaagggctaact
pyrE up-F	gttagttagcccttagtgactcgcgcattgatgtgaaaaccgc
pyrE up-R	tttttcattttaccgactgcttctcaagcaaatgtgtggcaa
pyrE down-F	acacatttgcttgagaagcagtcggtaaaatgaaaaatcca
pyrE down-R	cagctatgaccatgattacgccagcccgactttctaacaacacc

ribCs	ttagcccttagtgactcggactacgttcgcacgttagcggta
ribCm2	tcgttcttccgcggcgttcatcgcccaccaccgttccttc
ribCm1	gaaggaacggtggtgggcgatgaacgccgcggaagaacga
ribCa	tgaccatgattacgccaagccggactaataccgctccgtttgc
pUB31s	aacggagcggtattagtccggcttggcgtaatcatggtcat
pUB31a	ccgctaacgtgcgaacgtagtccgagtcactaagggctaactaa
purA down-F	tgtttgaagatgcgaaagcgtacacgact
purA down-R	ctatgaccatgattacgccaccagcaattctctcgtgctgaac
purA up-F	agttagcccttagtgactcgagtttcggagatttcccgttct
purA up-R	cgctttcgcatcttcaaacacttcgcggtcaag
purR up-F	gttagcccttagtgactcggaagcaccagcttcttcaagttcttc
purR up-R	agcagacgggatgctgtcttccgttgatgtgaaaga
purR down-F	cggaagacagcatcccgtctgcttcctgtct
purR down-R	gctatgaccatgattacgccatattccagtagcggcagggctg
ccpN up-F	agttagcccttagtgactcgcgacaaaaacgatcggcctc
ccpN up-R	tcgtcaaagatcatggcccgtgatgcgatgccggtagtgc
ccpN down-F	gcactaccggcatcgcatcacgggccatgatctttgacga
ccpN down-R	ctatgaccatgattacgccagacgaacaagtgacggaacg
ldh up-F	ttagttagttagcccttagtgactcggccgccaaccttgtttttct
ldh up-R	tgtttcatttagcgtcacatcgccctctgccttat
ldh down-F	aggcagagggcgatgtgacgctaaatgaaacagaacaa
ldh down-R	gaaacagctatgaccatgattacgccataaaaccaggcagactcca
$purR_{Gtg}$-F	gaaacacctgatgggtttgc
$purR_{Gtg}$-R	ctatgaaattaaggcgcagc
$purA_{Gtg}$-F	gggcaaacaaaatcggcacg
$purA_{Gtg}$-R	ggtcgccgatttcatcatgc
$ccpN_{Gtg}$-F	gtaaaactggctcgctgctg
$ccpN_{Gtg}$-R	caaacatcaggcagaaagcg
ldh_{Gtg}-F	atacttgataaagattgtgaaat
ldh_{Gtg}-R	catcctaaacacagttaagc
qPCR gap-F	cgcggcgcaaatctttatga
qPCR gap-R	caacgcttcttgcacaacga
qPCR prs-F	cgtggtctggagaaacgaca
qPCR prs-R	gtgcttccgccaaaacgatt
qPCR purE-F	gcccatcgtacgccagatta
qPCR purE-R	tgacgggaagcgtcgtttta
qPCR purF-F	ggaaccgggagagcttatcg
qPCR purF-R	gcgacggaaatgctggaatc
qPCR purA-F	ggggcgatgaaggaaaagga
qPCR purA-R	cattccgtttccgatgacgc

qPCR guaB-F	tcatcgtatcggctgtcacg
qPCR guaB-R	gtcgaaaagttgccgcttgt
qPCR pcka-F	tcggcaaagaaggggatgtc
qPCR pcka-R	catgctcgtcatcgccaatg
qPCR gapB-F	acaagcggctccatcgtaaa
qPCR gapB-R	tcgatcattccgacgacgac
